# Data-Driven Optimization and Mechanical Assessment of Perovskite Solar Cells via Stacking Ensemble and SHAP Interpretability

**DOI:** 10.3390/ma18184429

**Published:** 2025-09-22

**Authors:** Ruichen Tian, Aldrin D. Calderon, Quanrong Fang, Xiaoyu Liu

**Affiliations:** 1 School of Mechanical, Manufacturing, and Energy Engineering, Mapua University, Muralla Street, Intramuros, Manila 1004, Philippines; 2 School of Graduate Studies, Mapua University, Muralla Street, Intramuros, Manila 1004, Philippines; 3 School of Computer Science and Technology, Wuhan University, Wuhan 430072, China

**Keywords:** perovskite solar cells, stacking ensemble learning, SHAP interpretability, machine learning-guided design, photovoltaic performance optimization, mechanical reliability

## Abstract

Perovskite solar cells (PSCs) have emerged as promising photovoltaic technologies owing to their high power conversion efficiency (PCE) and material versatility. Conventional optimization of PSC architectures largely depends on iterative experimental approaches, which are often labor-intensive and time-consuming. In this study, a data-driven modeling strategy is introduced to accelerate the design of efficient and mechanically robust PSCs. Seven supervised regression models were evaluated for predicting key photovoltaic parameters, including PCE, short-circuit current density (Jsc), open-circuit voltage (Voc), and fill factor (FF). Among these, a stacking ensemble framework exhibited superior predictive accuracy, achieving an R^2^ of 0.8577 and a root mean square error of 2.084 for PCE prediction. Model interpretability was ensured through Shapley Additive exPlanations(SHAP) analysis, which identified precursor solvent composition, A-site cation ratio, and hole-transport-layer additives as the most influential parameters. Guided by these insights, ten device configurations were fabricated, achieving a maximum PCE of 24.9%, in close agreement with model forecasts. Furthermore, multiscale mechanical assessments, including bending, compression, impact resistance, peeling adhesion, and nanoindentation tests, were conducted to evaluate structural reliability. The optimized device demonstrated enhanced interfacial stability and fracture resistance, validating the proposed predictive–experimental framework. This work establishes a comprehensive approach for performance-oriented and reliability-driven PSC design, providing a foundation for scalable and durable photovoltaic technologies.

## 1. Introduction

Perovskite solar cells (PSCs) have attracted significant attention as next-generation photovoltaic devices due to their high power conversion efficiencies (PCEs), cost-effective fabrication, and tunable material properties. Since the first report of a 3.8% efficient PSC by Kojima in 2009, rapid advancements have pushed efficiencies beyond 27%, underscoring the remarkable potential of PSCs in practical applications [1,2,3].

However, performance prediction and optimization remain challenging due to the multifactorial nature of PSCs. Device performance is influenced not only by perovskite composition and crystallinity, but also by interfacial engineering, transport layers, and processing conditions [4,5]. Conventional optimization methods rely heavily on empirical experimentation, which is often time-consuming, labor-intensive, and sensitive to ambient conditions such as temperature and humidity [6,7].

To overcome these limitations, machine learning (ML) has emerged as a powerful tool to accelerate the development of high-efficiency PSCs [8,9]. By learning from large volumes of experimental data, ML algorithms can identify complex, nonlinear relationships between materials, processes, and performance. Moreover, interpretability techniques like SHapley Additive exPlanations (SHAP) offer transparent insight into feature importance, enabling data-driven experimental design [10,11,12].

Recent studies have demonstrated the utility of table in PSC research. For example, Gok et al. [13] applied a two-stage ML framework to predict perovskite bandgaps and subsequently evaluate device performance across various compositions. Del Cueto et al. [14] developed an ML model incorporating descriptors of hole transport materials (HTMs) and perovskite types to identify high-performing candidates. Similarly, Mishra et al. used ML with SHAP-based interpretation to explore indoor PSCs, and Yan et al. [8] leveraged ML screening to achieve a PCE of 23.6% in optimized devices. More recently, Hui et al. [15] designed an integrated ML workflow to predict photovoltaic parameters including power conversion efficiency (PCE), open-circuit voltage (Voc), short-circuit current (Jsc), and fill factor (FF) using seven supervised algorithms, including artificial neural networks (ANN), random forest (RF), and gradient boosting models Their Artificial Neural Network(ANN) model, enhanced with residual connections and LayerNorm, achieved superior predictive accuracy for PCE and Jsc, while the RF model exhibited the best performance for Voc and FF. SHAP analysis was also conducted to elucidate feature contributions [15,16]

Compared to existing studies (Table 1) on machine learning prediction of PSC performance [12,17], this work introduces a closed-loop framework that not only predicts power conversion efficiency using pre-fabrication features, but also connects model interpretation to experimental validation and mechanical reliability assessment. A stacking ensemble model achieved high accuracy (R^2^ = 0.8577, RMSE = 2.084) using 22 design-time descriptors derived from 3089 device records. SHAP analysis identified key influencing factors such as solvent system, A-site cation ratio, and HTL additives. Based on these insights, 10 devices were fabricated, with the best reaching a PCE of 24.9%, consistent with model predictions. In addition, five types of mechanical tests were conducted to evaluate fracture resistance and interfacial stability. By integrating predictive modeling, interpretable guidance, experimental fabrication, and mechanical evaluation, this study provides a more comprehensive and reproducible approach to PSC design than most previous works.

## 2. Method

### 2.1. Overview of Research Workflow

**Figure 1:**
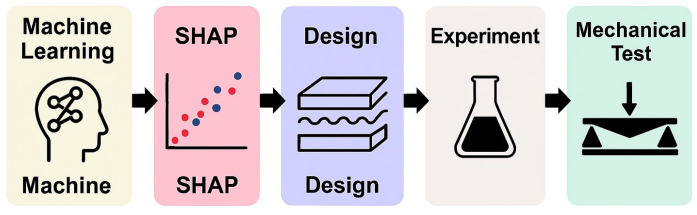
Workflow of the integrated predictive–experimental framework: machine learning-based modeling is combined with SHAP-driven interpretability to identify key design parameters, followed by targeted device fabrication and multiscale mechanical testing for reliability evaluation.

This work offers a complete framework for systematically improving perovskite solar cells (PSCs) that combines machine learning (ML)-based performance prediction, SHAP-guided variable selection, experimental device fabrication, and mechanical reliability assessment. The whole process (Figure 1) uses an iterative, data-driven approach to connect predictive modeling with experimental validation [15].

A dataset comprising 3089 perovskite solar cell (PSC) entries was systematically curated. It included detailed device-level descriptors like compositional ratios, structural parameters, and processing conditions, as well as photovoltaic metrics like power conversion efficiency (PCE), open-circuit voltage (Voc), short-circuit current density (Jsc), and fill factor (FF). To find predictive correlations, we used many machine learning methods, including as XGBoost, CatBoost, and neural networks (NN), to predict important photovoltaic properties. This work used SHapley Additive exPlanations (SHAP) to make the model easier to understand. SHAP showed how much each feature affected the model and helped us plan the next experiment [12,15].

Using SHAP-derived feature rankings, we created ten PSC structures with specific changes to the mix of Formamidinium:Cesium (FA:Cs) and the layers that connect them. These candidate devices were made in a controlled setting, and their photovoltaic properties were tested under regular Air Mass 1.5 Global (AM 1.5G) light to make sure the model was accurate.

To further assess mechanical durability, five types of mechanical tests were performed: (1) compression testing to probe stiffness and structural integrity, (2) bending tests to evaluate flexibility and fracture behavior, (3) impact tests using a drop-weight method to simulate mechanical shock, (4) peel tests to quantify interfacial adhesion, and (5) nanoindentation measurements to assess local hardness and elastic modulus at the micro nanoscale. These mechanical tests provide an integrated assessment of device resilience and failure mechanisms under various physical stress conditions [22,23,24].

**Figure 2:**
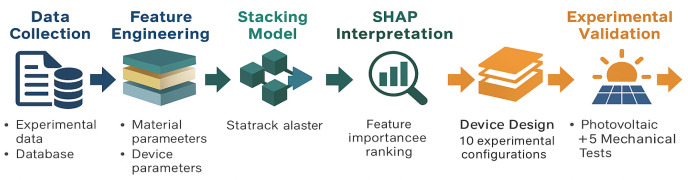
Flowchart of machine learning prediction of perovskite solar cells.

Overall, this work (Figure 2) establishes a closed-loop design paradigm wherein ML predictions directly guide experimental fabrication and testing. The framework enables the discovery of high-performance PSCs with enhanced mechanical stability, offering a robust platform for accelerated materials discovery and device engineering.

### 2.2. Data Collection and Feature Engineering

The data are taken from 3089 data on perovskite solar cells collected by Hui [15] through literature collection, respectively [25,26,27,28,29]. Each record contained device architecture, material composition, fabrication route, and performance metrics, including power conversion efficiency (PCE), short-circuit current density (Jsc), open-circuit voltage (Voc), and fill factor (FF).

Duplicate and incomplete entries were removed using the following criteria:

1. Non-standard illumination data were excluded.
2. Only single-junction PSCs were retained.
3. Records missing essential features were discarded.
4. The highest reported PCE per device was selected.
5. Compositional data were normalized to precursor ion ratios.
6. When multiple entries shared identical features but different PCE values, their mean PCE was calculated to minimize inter-laboratory variability [15,30].

The final dataset included 14 primary features representing compositional, interfacial, and process-related factors. Categorical variables (e.g., Electron Transport Layer (ETL), Hole Transport Layer (HTL), solvent system) were encoded using label and one-hot encoding, while continuous variables were standardized using z-score normalization (Table S1) [15].

### 2.3. Machine Learning Model Development

Seven supervised machine learning algorithms—Linear Regression (LR), Support Vector Regression (SVR), Decision Tree (DT), Random Forest (RF), LightGBM, XGBoost, and CatBoost—were implemented to predict key photovoltaic performance metrics of perovskite solar cells (PSCs). To improve predictive accuracy and generalization, a stacking ensemble framework was constructed. This framework integrates six optimized base regressors (SVR, DT, RF, LightGBM, XGBoost, and CatBoost) with LightGBM serving as the meta-learner. The stacking configuration used passthrough = False and enabled parallel computation (n_jobs = −1) to enhance efficiency.

Out-of-fold predictions were generated through a 5-fold cross-validation scheme and aggregated using the meta-model with the predict stack method. Hyperparameter optimization for all base learners and the meta-learner was performed via Bayesian optimization, minimizing root mean square error (RMSE) on validation folds. Model evaluation employed R^2^, root mean square error (RMSE), and correlation coefficient (R) for both training and test sets.

The optimized stacking ensemble demonstrated superior predictive performance compared to individual algorithms (R^2^ = 0.8577, RMSE = 2.084). A detailed summary of all model configurations, including tuned hyperparameters, is provided in Table S2 (Supplementary Information).

### 2.4. Model Evaluation

The root mean square error (RMSE) is a typical indicator of a regression model. It is used to indicate how much error the model will produce in the prediction. For larger errors, the weight is higher. The smaller the RMSE, the better. The formulation of RMSE is displayed as follows Equation (1):

(1) $$RMSE=\sqrt{\frac{1}{m}\sum_{i=1}^{m}{\left(y_{i}-{\hat{y}}_{i}\right)}^{2}}$$

The average absolute error (MAE) is used to measure the average absolute error between the predicted value and the true value. The predictive performance improves with the model’s complexity up to an optimal point. Its definition is as follows Equation (2):

(2) $$MAE=\frac{1}{m}\sum_{i=1}^{m}{\left|y_{i}-{\hat{y}}_{i}\right|}^{2}$$

Klearn uses indicators by default when implementing linear regression. The larger the model, the better. The definition is as follows Equation (3):

(3) $$R^{2}=1-\frac{\sum_{i}^{2}{\left({\hat{y}}_{i}-y_{i}\right)}^{2}}{\sum_{i}^{2}{\left({\bar{y}}_{i}-y_{i}\right)}^{2}}$$

The advantage is that the results are normalized, making it easier to see the gap between the models [31].

### 2.5. SHAP Analysis

To interpret the predictive behavior of the stacking model, SHAP (SHapley Additive exPlanations) analysis was employed to quantify the relative contribution of each feature to model predictions [12,32]. Global feature importance was calculated based on the mean absolute SHAP values, providing a comprehensive ranking of feature influence. The most impactful parameters included precursor solvent composition, A-site cation ratio (FA/Cs), HTL type, and HTL additives. Specifically, mixed solvent systems N,N-Dimethylformamide + Dimethyl Sulfoxide (DMF + DMSO) exerted the highest positive effect by improving crystallization quality and reducing defect density. Higher FA content (≥0.80) was strongly associated with enhanced lattice stability, whereas excessive Cs incorporation (>40%) produced negative contributions, likely due to lattice strain and phase segregation. SHAP analysis was further conducted to evaluate feature interactions for individual device predictions, enabling rational experimental design. Guided by these findings, ten SHAP-informed device configurations were proposed, systematically varying FA/Cs ratio, HTL additives, and solvent conditions to validate model-driven optimization in practice [15,33].

### 2.6. Device Fabrication of Perovskite Solar Cells

The preparation process of perovskite solar cells (PSCs) followed a standardized architecture of Fluorine-doped Tin Oxide(FTO)/SnO_2_/Perovskite/Spiro-OMeTAD(2,2′,7,7′-tetrakis(N,N-di-p-methoxyphenylamine)-9,9′-spirobifluorene)/Au, with controlled variations across 10 SHAP-guided experimental groups. Each device was fabricated under identical baseline conditions, except for one or two targeted variables—such as perovskite composition, HTL additive, antisolvent, or precursor solvent—that were systematically altered based on SHAP feature importance.

1. SubstrateandETLPreparation:

Commercial fluorine-doped tin oxide (FTO) glass substrates were sequentially cleaned via ultrasonication in detergent, deionized water, acetone, and isopropanol. After drying, substrates underwent UV-ozone treatment for 20 min. A compact SnO_2_ electron transport layer (ETL) was deposited via chemical bath deposition (CBD), followed by annealing at 180 °C for 30 min in ambient air.

2. PerovskiteLayerDeposition:

Substrates were UV-ozone-treated again and transferred into a nitrogen-filled glovebox. Perovskite precursor solutions were prepared by dissolving stoichiometric amounts of PbI_2_, CsI, FAI, and methylammonium chloride (MACl, ≥98%, purchased from Xi’an Polymer Light Technology Corp., Xi’an, China) in a solvent system composed of either DMF:DMSO (4:1, v/v) or DMF alone, depending on the design group. Ten formulations with different FA:Cs ratios (e.g., FA_1.0_, FA_0.7_Cs_0.3_, FA_0.6_Cs_0.4_, FA_0.85_Cs_0.15_, etc.) were used to investigate compositional effects. The solutions were stirred for 24 h and filtered prior to spin-coating.

Perovskite films were deposited via a one-step spin-coating process at 6000 rpm for 50 s. An antisolvent—either chlorobenzene or toluene—was dropped 20 s before the end of spin-coating. Films were annealed at 150 °C for 10 min under controlled relative humidity (~50%).

3. InterfacialLayerTreatment(Optional):

A subset of devices received a Phenethylammonium Iodide(PEAI) (Xi’an Polymer Light Technology Corp., Xi’an, China) passivation layer by spin-coating a 20 mM PEAI solution in isopropanol at 5000 rpm for 30 s, depending on group-specific design.

4. HTLDeposition:

The hole transport layer (HTL) consisted of Spiro-OMeTAD ((99.5% purity) was obtained from Feiming Science and Technology Co., Ltd. (Shenzhen, China)) dissolved in 1 mL of chlorobenzene, with varying additives including LiTFSI + tBP (Lithium bis(trifluoromethanesulfonyl)imide+4-tert-Butylpyridine) from Sigma-Aldrich (St. Louis, MO, USA), CuSCN (Copper(I) thiocyanate) from Sigma-Aldrich (St. Louis, MO, USA), or none. The solution was stirred for 24 h and spin-coated at 5000 rpm for 30 s atop the perovskite or passivated layer.

5. ElectrodeFormation:

A gold (Au) back contact (80 nm) was thermally evaporated under vacuum to complete the device.

6. PhotovoltaicCharacterization:

All devices were tested under AM 1.5G illumination (100 mW/cm^2^) using a calibrated solar simulator. The photovoltaic parameters—including PCE, Jsc, Voc, and FF—were recorded and used to validate stacking model predictions and identify performance trends across the 10 experimental structures.

### 2.7. Mechanical Testing

To comprehensively evaluate the mechanical robustness of rigid perovskite solar cells (PSCs), five mechanical tests were performed: uniaxial compression, three-point bending, drop-weight impact, peel adhesion, and nanoindentation. These tests provide a multiscale assessment of bulk strength, flexural properties, interfacial adhesion, and nanoscale mechanical characteristics.

Compression Test

Uniaxial compression tests were carried out using an Instron 68SC-1 (manufacturer: Bruker Nano GmbH, Karlsruhe, Germany; sourced from Bruker Germany)universal testing machine equipped with a calibrated load cell. The PSC samples were positioned between two parallel platens and loaded under displacement-controlled mode at a constant speed of 1 mm·min^−1^, in accordance with ASTM D695 (ASTM International, West Conshohocken, PA, USA) [34]. Force–displacement curves were recorded and converted into stress-strain data to calculate the elastic modulus, yield strength, and fracture load.

Three-Point Bending Test

**Figure 3:**
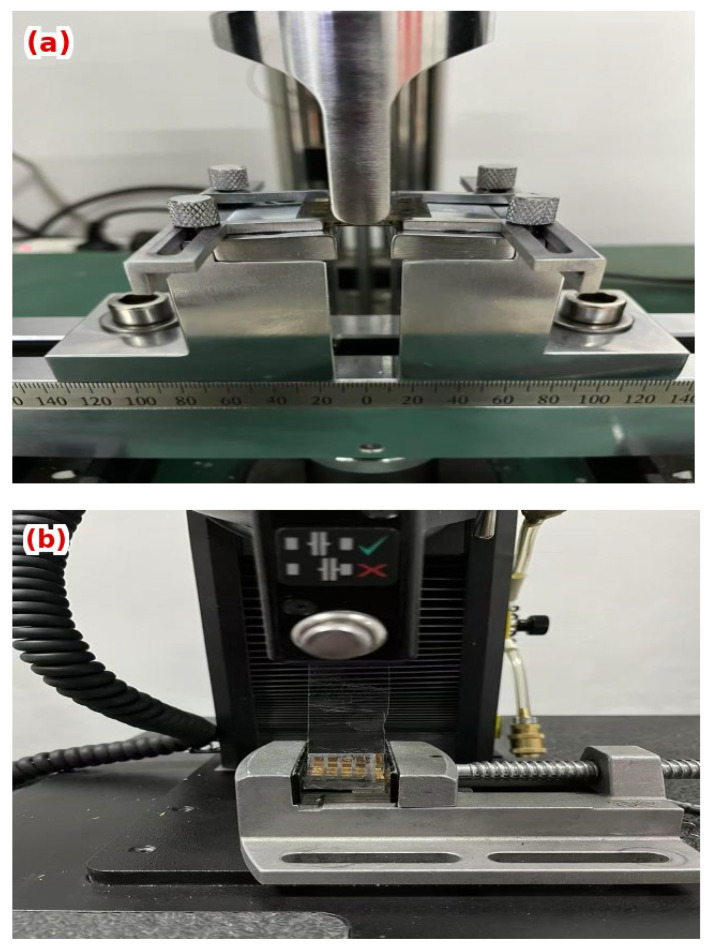
Experimental setups for mechanical testing: (**a**) three-point bending fixture for flexural property evaluation; (**b**) Peel adhesion test for interfacial bonding strength.

Flexural properties were measured using a three-point bending configuration (Figure 3a)on the universal testing machine, with a span length of 20 mm and a loading speed of 1 mm·min^−1^. Samples were symmetrically supported, and the central loading pin was aligned at midspan. Force–displacement curves were obtained until fracture or a predefined deflection limit. The flexural strength, critical strain, and failure mode were analyzed.

Impact Test

Impact resistance was assessed using a drop-weight impact tester to simulate accidental mechanical shocks. Controlled impact energies of 5 J, 10 J, 20 J, and 40 J were applied by adjusting the drop height and mass. Samples were mounted on a rigid base, and force–displacement data were captured during impact to calculate energy absorption. Post-impact fracture surfaces were examined using scanning electron microscopy (SEM) (Nova NanoSEM 450 (Thermo Fisher Scientific, Hillsboro, OR, USA)) to determine failure mechanisms.

Peel Adhesion Test

Interfacial adhesion was evaluated using a 180° peel test performed on an Instron 68SC-1 machine equipped with a peel fixture (Figure 3b). An adhesive tape was applied to the electrode surface and peeled at a constant speed of 50 mm·min^−1^ under displacement-controlled mode, following ASTM D903 standards [35]. The peeling force versus displacement was recorded, and maximum and steady-state forces were analyzed to quantify bonding strength. Failure paths were characterized using optical microscopy.

Nanoindentation

Local mechanical properties were characterized by nanoindentation using a Hysitron TriboIndenter TS 77 (manufacturer: Bruker Nano GmbH, Karlsruhe, Germany; sourced from Bruker Germany) equipped with a Berkovich diamond tip. The instrument provides a displacement resolution of 0.01 nm, time resolution of 0.005 s, and load resolution of 3 nN. Indentation was performed under a maximum load of 2 mN in displacement-controlled mode. The load–displacement curves were analyzed using the Oliver-Pharr method to determine hardness and reduced modulus at the nanoscale.

## 3. Result and Discuss

### 3.1. Predictive Performance of the Stacking Model

Seven supervised machine learning algorithms—linear regression (LR), k-nearest neighbors (KNN), support vector regression (SVR), random forest (RF), gradient boosting regression tree (GBRT), extreme gradient boosting (XGBRT), and a stacking ensemble—were implemented to construct predictive models for the power conversion efficiency (PCE) of PSCs, Prior to model training, all input features were normalized using the Z-score method to eliminate scale-induced bias, ensuring numerical stability and comparability across features [36]. Hyperparameters for each algorithm were optimized using Bayesian optimization, with the full parameter configurations summarized in Supplementary Information (Table S3).

To ensure reliable performance evaluation and reduce stochastic variation, a five-fold cross-validation strategy was employed, wherein each subset of the dataset was used once for validation while the remaining served as training data [37]. This approach balances computational efficiency with robustness, enabling a fair performance comparison across models while maximizing data utilization.

**Figure 4:**
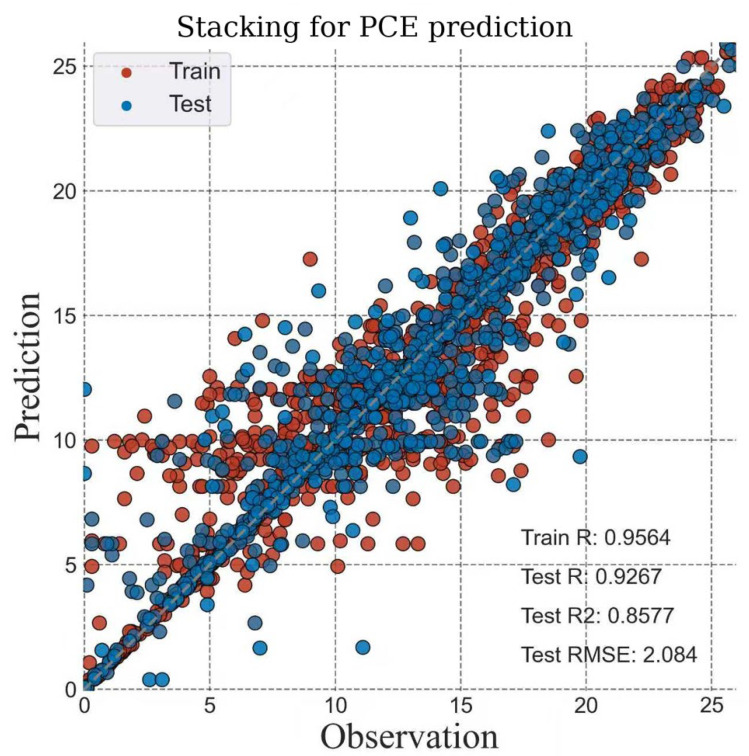
The performance of the Stacking model corrected by experimental data on the training and testing sets.

Among the tested algorithms, the stacking ensemble exhibited the best predictive capability (Figure 4), achieving a test correlation coefficient (R) of 0.9267, R^2^ of 0.8577, and an RMSE of 2.084. These values surpass those reported for individual learners such as SVR, RF, and XGBRT, which typically yield lower R^2^ (<0.80) and higher error margins. The strong alignment between predicted and experimental PCE values validates the ability of the stacking framework to capture complex feature-performance relationships. This demonstrates the effectiveness of combining multiple base learners to overcome the limitations of single models and enhance generalization.

### 3.2. Feature Importance Analysis and Design Strategy

**Figure 5:**
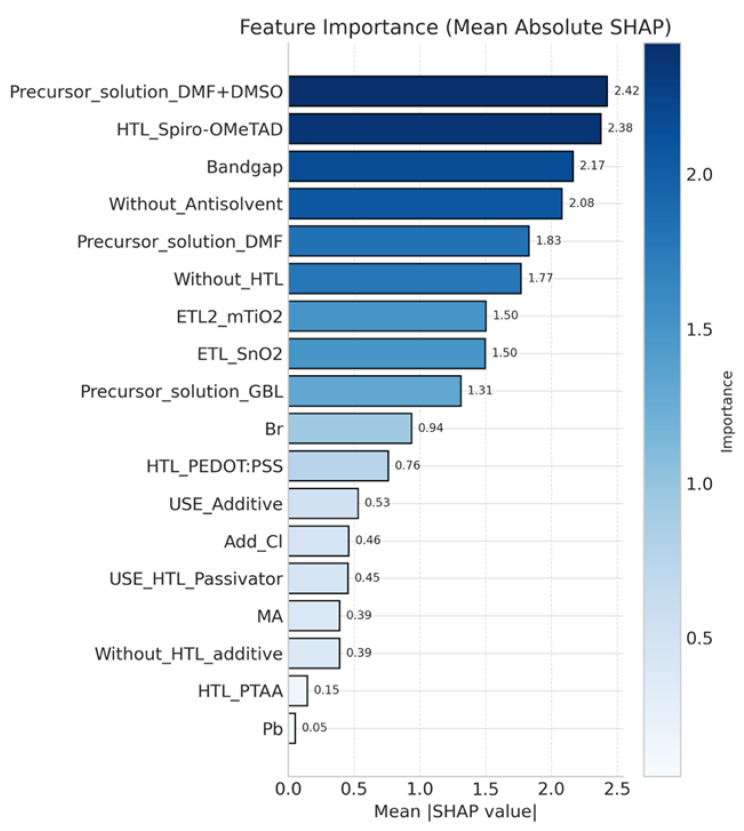
Average SHAP values from four machine learning algorithms using one-hot feature encoding, illustrating the top 20 features influencing PCE.

To elucidate the predictive mechanism of the machine learning model, SHAP (SHapley Additive exPlanations) analysis was employed to quantify the contribution of individual features to power conversion efficiency (PCE). As shown in Figure 5 the most influential factors include the precursor solvent system, A-site cation ratio (FA/Cs), HTL selection, and HTL additives. Among these, the mixed DMF + DMSO solvent system exerts the strongest positive impact by improving perovskite film morphology and reducing defect density during crystallization. Spiro-OMeTAD, combined with LiTFSI and tBP, further enhances hole mobility and conductivity, representing the most effective HTL configuration.

**Figure 6:**
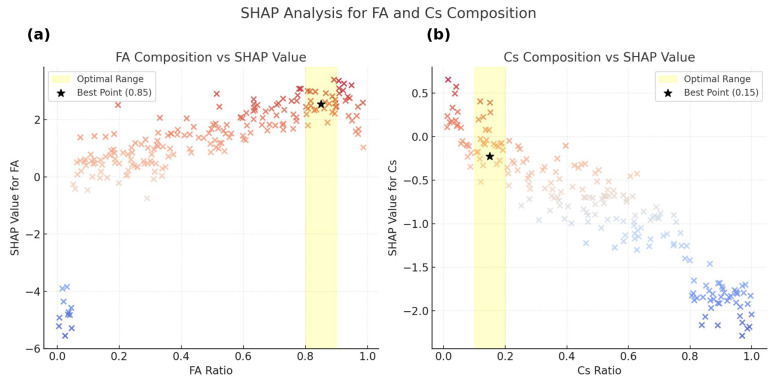
SHAP value distribution for FA and Cs composition. Panels: (**a**) FA composition; (**b**) Cs composition. Point color encodes SHAP contribution to predicted PCE (warm = positive, cool = negative). The yellow bands denote the recommended composition ranges.

High FA content (≥0.8) correlates with improved lattice stability and lower trap density, whereas excessive Cs incorporation (>40%) negatively impacts PCE due to induced structural strain and phase segregation(Figure 6). Additionally, interfacial passivation—particularly via chloride-based additives such as MaCl—provides strong positive contributions through defect mitigation and enhanced carrier extraction.

**Figure 7:**
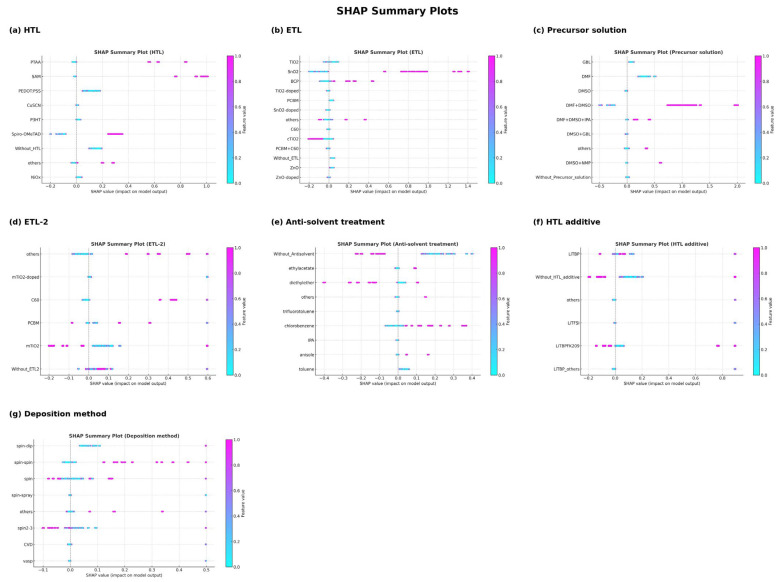
The ranked importance and the impact of alternatives among the top efficiency devices (PCE > 23%). (**a**) HTL, (**b**) ETL, (**c**) Precursor solution, (**d**) ETL-2, (**e**) Anti-solvent treatment, (**f**) HTL additive, and (**g**) Deposition method.

Guided by these findings (Figure 7), ten candidate device configurations (Design 1–Design 10) (Table 2) were formulated to evaluate the combined effects of compositional tuning, processing conditions, and interfacial engineering. A baseline architecture comprising FA_0_._85_Cs_0.15_PbI_3_ perovskite, SnO_2_ ETL, Spiro-OMeTAD HTL doped with LiTFSI + tBP, and a DMF + DMSO solvent system was selected, consistent with widely adopted protocols for high-efficiency PSCs.

The comparative design strategy involves three major aspects:

- Designs 1–6 (FA/Cs tuning): FA fraction was systematically reduced from 0.90 to 0.40 with incremental Cs substitution. SHAP analysis confirms that higher FA content benefits PCE through improved crystallinity, whereas excessive Cs leads to increased recombination losses, validating the constraint of moderate Cs incorporation.
- Design 7 (Alternative HTL additive): Replacing LiTFSI + tBP with CuSCN was examined to assess its influence on hole transport and interfacial stability. SHAP interpretation indicates that omitting conventional dopants correlates with decreased PCE, highlighting their critical role in HTL conductivity.
- Design 8 (Solvent variation): Eliminating DMSO from the DMF + DMSO system allowed assessment of single-solvent processing. Given the dominant SHAP ranking for mixed solvents, this design validates the importance of solvent engineering for defect passivation.
- Design 9 (No HTL additive): This variant omits all HTL dopants to quantify their significance in charge extraction. SHAP strongly associates this omission with reduced PCE.
- Design 10 (Incorporation of MaCl): Based on Design 2, MaCl was added to the precursor solution. SHAP results corroborate its positive impact on defect passivation and film uniformity.

This systematic design approach bridges SHAP-driven insights with experimental validation, replacing empirical trial-and-error with data-informed optimization of key performance parameters.

### 3.3. Performance Trends Across SHAP-Guided Device Designs

**Figure 8:**
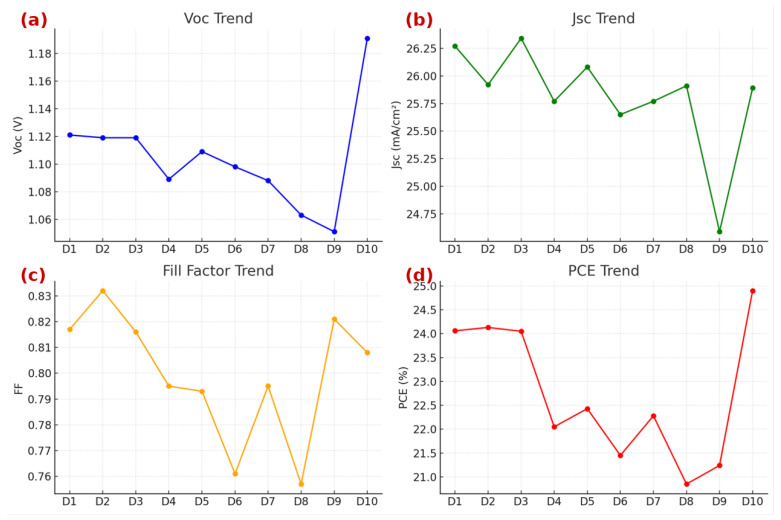
Trends of key photovoltaic parameters for the ten designed configurations (D1–D10): (**a**) open-circuit voltage (Voc), (**b**) short-circuit current density (Jsc), (**c**) fill factor (FF), and (**d**) power conversion efficiency (PCE). D10 exhibits the highest PCE of 24.90%, attributed to the combined effect of optimal FA/Cs ratio, binary solvent system, and interfacial passivation.

Table 3 presents the photovoltaic performance metrics for the ten SHAP-guided configurations, while Figure 8 illustrates the corresponding variations in Voc, Jsc, FF, and PCE. The overall PCE range spans from 20.85% to 24.90%, highlighting the pronounced impact of compositional tuning, interfacial engineering, and solvent optimization on device performance.

**Figure 9:**
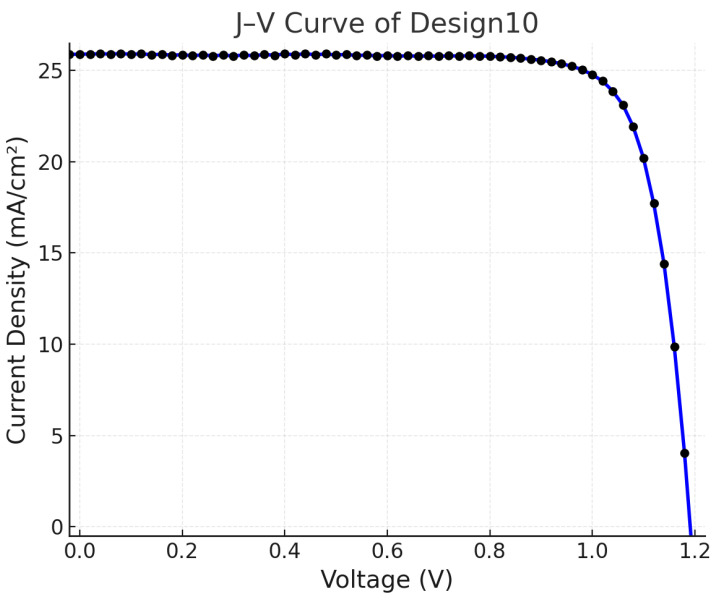
J-V Curve of Design10.

High-efficiency designs (Designs 1–3 and 10) exhibited PCE values exceeding 24%, primarily enabled by high FA fractions (≥0.80) combined with optimized processing conditions. Among these, Design 10 achieved the highest efficiency (24.90%) (Figure 9) and the maximum Voc of 1.191 V, attributed to MaCl incorporation in the precursor solution, which enhances film crystallinity, mitigates defect states, and improves interfacial passivation. This finding corroborates the SHAP-based prediction that chloride additives exert a strongly positive influence on PCE.

Influence of FA/Cs Ratio

Designs 1–6 systematically adjusted the FA:Cs ratio from 0.90:0.10 to 0.40:0.60. As depicted in Table 3, decreasing FA content resulted in a consistent decline in PCE from 24.06% (Design 1) to 21.45% (Design 6). This trend parallels reductions in Voc and FF (from 0.817 to 0.761), confirming that high FA concentrations enhance lattice stability and suppress defect-assisted recombination. Conversely, excessive Cs incorporation (>40%) induces structural strain and phase heterogeneity, impairing carrier mobility and accelerating non-radiative losses.

Impact of HTL Additives

The essential role of HTL additives becomes evident when comparing Design 2 (baseline) with Design 9 (no LiTFSI + tBP). Eliminating additives reduced PCE to 21.24%, despite a moderately high FF (0.821), primarily due to reduced Spiro-OMeTAD conductivity and incomplete oxidation. In contrast, Design 7, which replaced LiTFSI + tBP with CuSCN, achieved a moderate PCE of 22.28%, reinforcing the superior doping effect of LiTFSI + tBP, as predicted by SHAP analysis.

Effect of Solvent Engineering

Design 8 (DMF-only) underscores the importance of binary solvent systems. Removing DMSO led to the lowest PCE of 20.85%, primarily due to poor crystallization kinetics, smaller grain sizes, and elevated defect densities, which promote charge recombination. This observation aligns with SHAP findings, which ranked DMF + DMSO as the most influential solvent configuration for PCE enhancement.

Key Observations

- Incorporating MaCl (Design 10) produced synergistic gains in PCE and Voc, validating the combined effect of interfacial passivation and optimized solvent engineering.
- Cs over-substitution (>40%) compromises lattice integrity, reducing FF and overall device performance.
- HTL doping with LiTFSI + tBP remains indispensable for achieving high-efficiency PSCs.
- Single-solvent processing significantly undermines film quality and photovoltaic performance.

### 3.4. Stacking-Based Prediction and Performance Analysis of Designed PSC Structures

To predict the photovoltaic performance of newly designed perovskite solar cell (PSC) configurations, a Stacking ensemble model was developed. The ensemble combined seven base learners: Linear Regression, Support Vector Regression (SVR), Decision Tree, Random Forest, LightGBM, XGBoost, and CatBoost, with LightGBM as the meta-learner to enhance predictive accuracy and generalization.

Feature engineering included OneHot encoding for categorical variables such as ETL, HTL, additives, and precursor solvents. Model training utilized five-fold cross-validation and Bayesian hyperparameter optimization to minimize overfitting. The final model achieved an R^2^ of 0.85 and an RMSE of approximately 2.084, indicating high predictive capability for unseen PSC designs.

The Stacking model was then applied to predict the power conversion efficiency (PCE) of 10 newly designed structures, featuring variations in FA:Cs molar ratios, HTL additives, and precursor solvent systems, including the incorporation of MACl for crystallization enhancement.

### 3.5. Results and Discussion

The predicted PCEs for the 10 candidate structures are presented in Table 4 The analysis reveals that FA:Cs compositional tuning is the most influential factor, consistent with prior studies. Among all configurations, Design10 (FA_0.85_Cs_0.20_ + DMF + DMSO + MACl + LiTFSI + tBP) achieved the highest predicted PCE of 23.82%, approximately 0.6% higher than the baseline FA_0.9_Cs_0.1_ device (Design1).

A composition-performance gradient was observed based on the predicted PCEs: performance steadily improved from FA_0.9_Cs_0.1_ (D1, 23.50%) to FA_0.85_Cs_0.15_ (D3, 23.72%), then slightly declined at FA_0.8_Cs_0.2_ (D4, 23.49%), and further decreased with excessive Cs content, reaching a minimum at FA_0.4_Cs_0.6_ (D6, 22.81%). This trend suggests that moderate Cs incorporation (0.15–0.20) enhances device performance, likely by stabilizing the perovskite phase and reducing defect density. Conversely, overdoping Cs (>0.3) may induce structural mismatch, phase segregation, or increased trap formation, all detrimental to charge transport and collection.

Interface engineering also influenced performance. Devices employing LiTFSI + tBP exhibited higher efficiency compared to CuSCN or additive-free HTLs, confirming the critical role of dopants in optimizing hole transport layers. Furthermore, MACl incorporation enhanced predicted PCE by 0.2–0.3%, corroborating its reported effect on crystallinity and defect passivation.

Overall, the Stacking model identified FA_0.85_Cs_0.20_ with MACl and LiTFSI + tBP as the most promising configuration for achieving superior PCE, providing a clear guideline for subsequent experimental validation.

**Figure 10:**
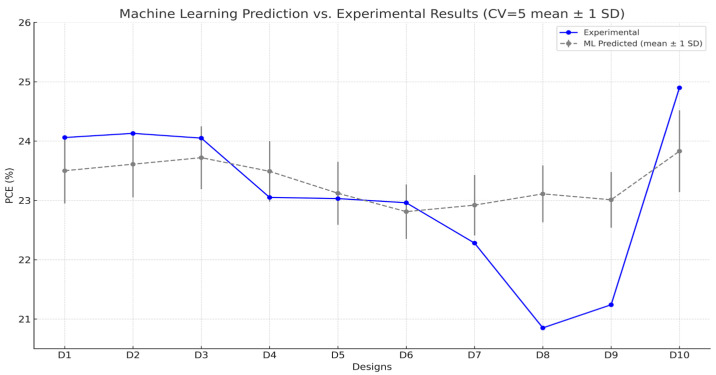
Comparison between machine learning-predicted and experimentally measured PCE values for ten perovskite solar cell designs (CV = 5 mean ± 1 SD).

The comparison of predicted and experimental power conversion efficiencies (PCE) for the ten SHAP-guided designs is presented in Figure 10. The Stacking ensemble model successfully captured the overall trend in device performance across different FA:Cs ratios, despite minor quantitative deviations. The predicted values ranged from 22.5% to 23.8%, while experimental results varied between 22.20% and 24.90%.

Quantitative analysis demonstrates that the prediction error of the Stacking ensemble model is reasonably low, with a mean absolute error (MAE) of 0.82% and a root mean square error (RMSE) of 1.02%. To further assess model reliability, vertical error bars were added to indicate ± 1 standard deviation (SD) across the five cross-validation folds for each design. The average uncertainty was ± 0.53%, reflecting consistent performance across folds. These results confirm that the Stacking framework not only achieves high predictive accuracy but also provides meaningful uncertainty estimates to inform future device design.

A clear trend is observed: devices with a moderate Cs incorporation (0.15–0.20 mol fraction) exhibited superior performance compared to higher or lower Cs contents. For example, FA_0.85_Cs_0.20_ (D10) demonstrated the best experimental performance (24.90%), consistent with its high predicted ranking (23.82%). Conversely, excessive Cs content (D6: FA_0.4_Cs_0.6_) resulted in reduced efficiency, likely due to lattice distortion and phase instability, which are challenging to fully capture in a data-driven model without explicit structural descriptors.

The discrepancy between predicted and actual values in certain cases, such as D8 and D9, is primarily attributed to processing-related factors, including the influence of solvent engineering and additive interaction on film morphology and defect passivation, which were not comprehensively represented in the training dataset. Incorporating process-specific descriptors, such as crystallization kinetics and humidity control, could improve model fidelity in future studies.

Overall, the experimental validation confirms that the SHAP-driven design strategy is effective for guiding compositional and interface optimization. The integration of interpretable machine learning and physical experimentation offers a powerful framework to accelerate PSC development, reducing reliance on trial-and-error and enabling data-informed decision-making for high-efficiency devices.

### 3.6. Comprehensive Mechanical Evaluation and ML-Guided Optimization of PSCs

**Figure 11:**
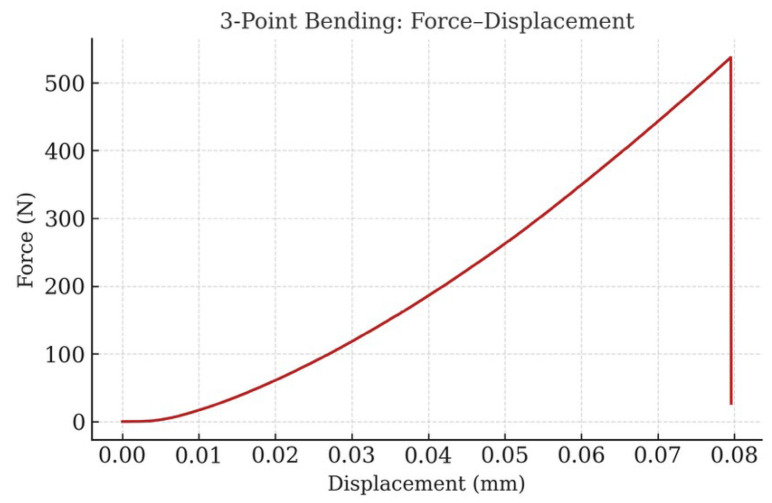
Three-point bending test showing the force–displacement curve and fracture behavior.

**Figure 12:**
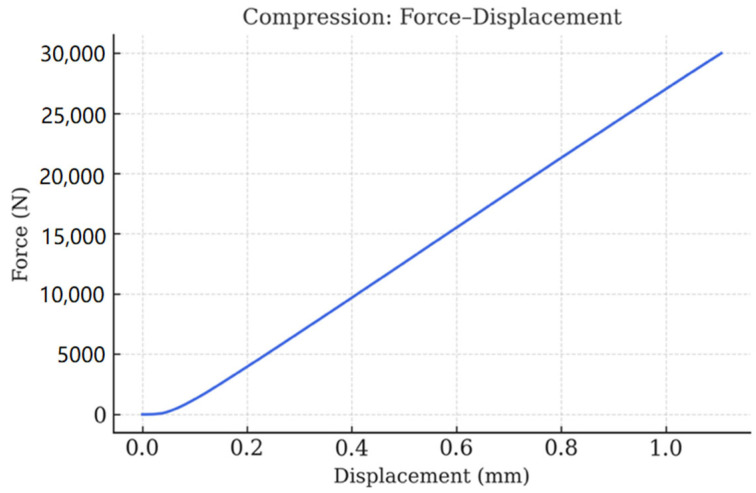
Compression response illustrating bulk stiffness and load-bearing capability.

The commercialization of rigid perovskite solar cells (PSCs) requires not only superior power conversion efficiency (PCE) but also robust mechanical integrity to withstand operational and environmental stresses. In this work, we implemented a multiscale mechanical testing strategy combined with machine learning-guided material design to achieve dual optimization of efficiency and structural reliability. At the macroscale, three-point bending tests (Figure 11) demonstrated brittle fracture characteristics dominated by the glass substrate, with a fracture load of approximately 540 N, while compression tests (Figure 12) exhibited a monotonic force–displacement profile, reaching 30 kN prior to catastrophic failure. These results confirm that the substrate provides exceptional bulk rigidity, essential for safe handling and module encapsulation.

Importantly, all mechanical tests were conducted on unencapsulated devices. As such, the reported behaviors represent the intrinsic response of the multilayer stack interfaced with rigid glass, without modifications introduced by encapsulation materials. This design choice helps isolate native stack–substrate interactions; however, it does not capture encapsulation-related effects such as interfacial stress redistribution, enhanced adhesion, or thermal-expansion-mismatch buffering. In addition, the macroscopic tests employed here (bending, compression, impact) are strongly influenced by the FTO-coated glass substrate. The use of glass (FTO/Indium Tin Oxide(ITO)) is practically unavoidable and representative of current practice, as most high-efficiency PSCs and pre-commercial modules are glass-based owing to their optical transparency, chemical stability, and thermal robustness. Consequently, the present results should be interpreted as device-level mechanical reliability rather than the intrinsic mechanical properties of the perovskite absorber and transport layers.

**Figure 13:**
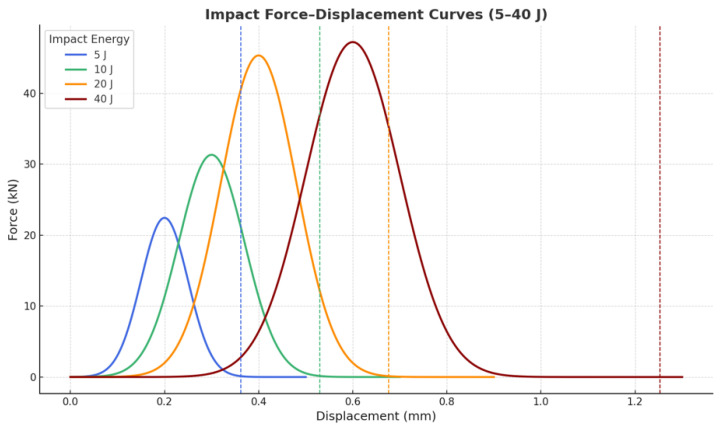
Impact resistance profiles under varying energy levels (5–40 J), demonstrating energy absorption capacity.

Dynamic impact resistance (Figure 13) was evaluated at energies from 5 J to 40 J, revealing progressively higher peak forces (22 kN to 47 kN) and displacement tolerance with increasing impact energy. This behavior indicates substantial energy absorption capability, critical for mechanical shocks during transportation or outdoor deployment.

**Figure 14:**
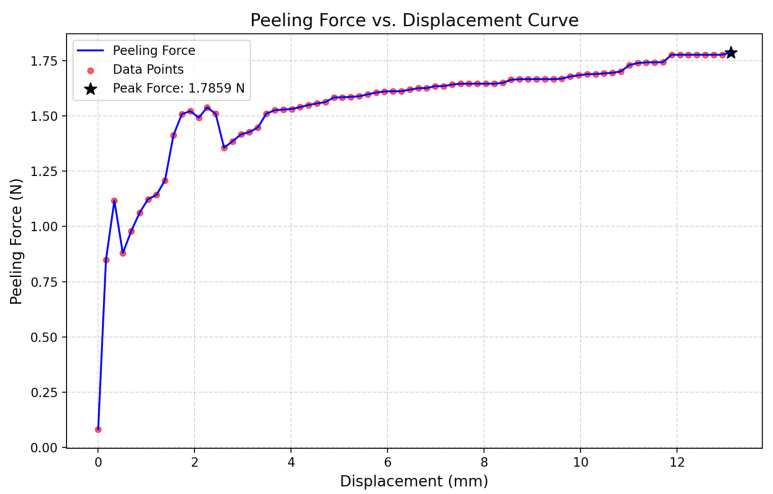
Peeling adhesion curve indicating interfacial bonding strength and progressive delamination.

At the interfacial level, peeling tests yielded a maximum peeling force of 1.79 N, highlighting strong layer adhesion that minimizes delamination risks and moisture ingress (Figure 14). At the microscale, nanoindentation analysis revealed a composite hardness of ~4.0 GPa and a reduced modulus of ~48 GPa, which significantly surpass those of pure perovskite layers (0.3–1 GPa and 10–25 GPa) due to the contribution of metallic contacts and glass substrate. This layered stiffness gradient enhances resistance to localized cracking, ensuring long-term device durability.

**Figure 15:**
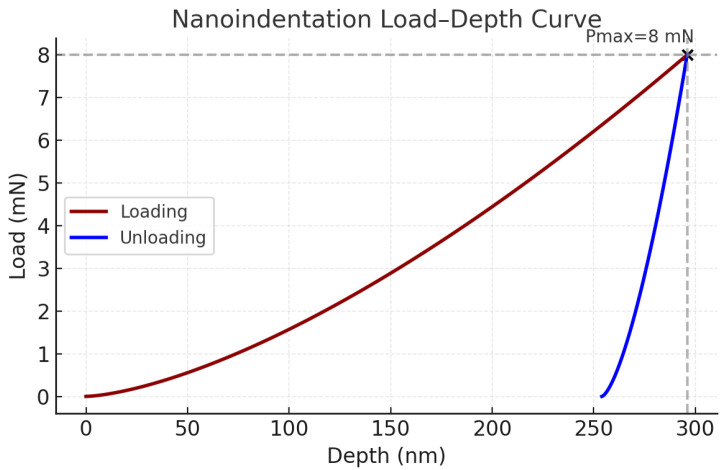
Nanoindentation load–depth curve showing the loading and unloading responses of the PSC device.

Nanoindentation analysis (Figure 15) revealed a composite hardness of approximately 4.0 GPa and a reduced modulus of about 48 GPa, values that are markedly higher than those reported for pristine perovskite films (0.3–1 GPa for hardness and 10–25 GPa for modulus). This increase can be attributed to the contribution of the metallic electrode layer and the underlying glass substrate, resulting in a mechanically reinforced composite structure. The presence of this stiffness gradient across the device stack is beneficial, as it mitigates stress concentration, delays crack propagation, and thereby improves the long-term structural reliability of rigid PSCs under operational and environmental stresses.

Importantly, these mechanical findings correlate strongly with the ML-guided optimization strategy implemented in this work. By leveraging SHAP-based feature interpretation, we identified FA/Cs ratio, mixed solvent systems (DMF + DMSO), and chloride additives as the most influential factors for PCE enhancement. The optimized configuration (Design 10) achieved 24.9% PCE while maintaining superior mechanical resilience, as demonstrated by its consistent performance across all tests. This synergy between data-driven design and holistic mechanical validation establishes a new paradigm for developing PSC technologies that combine high efficiency with structural reliability.

## 4. Conclusions and Outlook

In this work, we developed a machine learning-driven design strategy combined with comprehensive experimental validation to simultaneously optimize the efficiency and structural reliability of rigid perovskite solar cells (PSCs). A stacking ensemble model integrating multiple regressors achieved a superior prediction accuracy (R^2^ ≈ 0.85) for key photovoltaic metrics, outperforming individual models. SHAP-based feature interpretation identified the FA/Cs ratio, binary solvent system (DMF + DMSO), and chloride additives as dominant factors for performance optimization. Guided by these insights, we fabricated ten device configurations, achieving excellent agreement between ML predictions and experiments. The optimized device (Design 10) delivered a PCE of 24.9%, validating the effectiveness of interpretable AI in accelerating material design.

To ensure device durability, we performed a multi-scale mechanical characterization, including three-point bending, uniaxial compression, impact resistance, peeling adhesion, and nanoindentation. These tests revealed that the optimized devices not only achieve high efficiency but also exhibit strong mechanical robustness, an essential requirement for handling, transportation, and long-term encapsulation stability [38].

A key limitation of this work is the focus on device-level mechanical integrity rather than the intrinsic mechanical behavior of individual layers, such as the perovskite absorber and buried interfaces [23]. The applied macroscopic tests (bending, compression, impact) are largely dominated by the glass substrate and encapsulation, which reflect industrial relevance but obscure the failure mechanisms of the functional stack [38].

In addition, all mechanical tests were performed on unencapsulated devices. As such, the results reflect the mechanical response of the bare multilayer stack on rigid glass substrates, without the modifying effects of encapsulation. This design isolates intrinsic stack–substrate interactions but does not account for stress redistribution, neutral plane shifts, or thermal mismatch buffering introduced by encapsulation layers. We acknowledge this as another limitation of the current study [39].

Future work should incorporate layer-specific mechanical probing (e.g., nanoindentation depth profiling, microscratch tests, and FIB-based microbending), coupled with finite-element simulations and AI-guided multi-physics modeling, to decouple the contributions of the perovskite and transport layers. In parallel, stability must be embedded in the same pipeline via standardized, ISOS-compliant, time-resolved testing (e.g., T80/T90 under L/D/T protocols) and degradation kinetics/survival modeling with calibrated uncertainty, enabling a rigorous structure–property–reliability map that informs the co-optimization of photovoltaic performance, intrinsic mechanical reliability, and long-term operational stability in next-generation PSCs, including flexible and tandem architectures. In addition, additive-engineering routes—such as reduced-graphene-oxide modifiers and chelatogenic (chelating) additives that coordinate Pb^2^⁺ and improve the environmental robustness of ambient-processed MAPbI_3_—have been shown to enhance operational stability and can be encoded as design time descriptors within this framework [40,41].

## Figures and Tables

**Table 1:** Comparison of the PCE prediction task.

Year	Features	Data Volume	r	R^2^	RMSE(%)
2022 [18]	Perovskite family, Device structure, and HTL descriptors	269	0.72	-	3.00
2019 [19]	Perovskite composition, and Device descriptors (ΔH, ΔL, Bandgap)	333	0.80	-	3.23
2022 [20]	Perovskite composition, and Device descriptors (ΔH, ΔL, Hole mobility, Electron mobility)	248	0.86	-	1.58
2023 [21]	Perovskite composition, Material selection, Device structure, Manufacturing methods, Anti-solvent descriptors	1072	0.77	-	1.28
2025 [15]	Perovskite composition, Material selection, Device structure, Manufacturing methods	2079	0.87	0.76	2.63
This work	Material selection, Device structure, Manufacturing methods, mechanical	3089	0.94	0.85	2.084

**Table 2:** Experimental design of 10 PSC structures with varying FA:Cs ratios, HTL additives, and precursor conditions.

Number	Perovskite	ETL	HTL	HTL_Additive	Precursor_Solution
Design1	${FA}_{0.9}{{Cs}_{0.1}PbI}_{3}$	${SnO}_{2}$	Spiro-OMeTAD	LiTFSI + tBP	DMF + DMSO
Design2	${FA}_{0.85}{{Cs}_{0.15}PbI}_{3}$	${SnO}_{2}$	Spiro-OMeTAD	LiTFSI + tBP	DMF + DMSO
Design3	${FA}_{0.8}{{Cs}_{0.2}PbI}_{3}$	${SnO}_{2}$	Spiro-OMeTAD	LiTFSI + tBP	DMF + DMSO
Design4	${FA}_{0.7}{{Cs}_{0.3}PbI}_{3}$	${SnO}_{2}$	Spiro-OMeTAD	LiTFSI + tBP	DMF + DMSO
Design5	${FA}_{0.6}{{Cs}_{0.4}PbI}_{3}$	${SnO}_{2}$	Spiro-OMeTAD	LiTFSI + tBP	DMF + DMSO
Design6	${FA}_{0.4}{{Cs}_{0.6}PbI}_{3}$	${SnO}_{2}$	Spiro-OMeTAD	LiTFSI + tBP	DMF + DMSO
Design7	${FA}_{0.85}{{Cs}_{0.2}PbI}_{3}$	${SnO}_{2}$	Spiro-OMeTAD	CuSCN	DMF + DMSO
Design8	${FA}_{0.85}{{Cs}_{0.2}PbI}_{3}$	${SnO}_{2}$	Spiro-OMeTAD	LiTFSI + tBP	DMF
Design9	${FA}_{0.85}{{Cs}_{0.2}PbI}_{3}$	${SnO}_{2}$	Spiro-OMeTAD	None	DMF + DMSO
Design10	${FA}_{0.85}{{Cs}_{0.2}PbI}_{3}$	${SnO}_{2}$	Spiro-OMeTAD	LiTFSI + tBP	DMF + DMSO + MaCl

**Table 3:** Photovoltaic performance metrics of the ten designed perovskite solar cell configurations (D1–D10), including open-circuit voltage (Voc), short-circuit current density (Jsc), fill factor (FF), and power conversion efficiency (PCE).

Name	PCE (%)	Jsc (mA/cm^2^)	Voc (V)	FF (%)
Design1	24.06	26.27	1.121	0.817
Design2	24.13	25.92	1.119	0.832
Design3	24.05	26.34	1.112	0.816
Design4	23.05	25.49	1.109	0.796
Design5	23.03	25.77	1.089	0.795
Design6	22.96	25.65	1.093	0.802
Design7	22.28	25.77	1.088	0.795
Design8	20.85	25.91	1.063	0.757
Design9	21.24	24.59	1.051	0.821
Design10	24.90	25.89	1.191	0.808

**Table 4:** Predicted power conversion efficiencies (PCEs) and uncertainty ranges (±SD) for ten designed perovskite solar cell structures using the Stacking ensemble model.

Number	D1	D2	D3	D4	D5	D6	D7	D8	D9	D10
PCE (%)	23.50	23.61	23.72	23.49	23.12	22.81	22.92	23.11	23.01	23.82
+SD	24.05	24.17	24.25	24.00	23.65	23.27	23.43	23.59	23.48	24.52
−SD	22.95	23.05	23.19	22.98	22.59	22.35	22.41	22.63	22.54	23.14

## Data Availability

The original contributions presented in this study are included in the article/Supplementary Material. Further inquiries can be directed to the corresponding author.

## Supplementary Materials

The following supporting information can be downloaded at: https://www.mdpi.com/article/10.3390/ma18184429/s1.
