# Supplementary material for: Data-Driven Optimization and Mechanical Assessment of Perovskite Solar Cells via Stacking Ensemble and SHAP Interpretability
Source: Materials (Basel). 2025 Sep 22;18(18):4429. doi: 10.3390/ma18184429 (PMC12471739; doi:10.3390/ma18184429)
Supplement: Supplementary file 1 [file materials-18-04429-s001.zip › materials-3826319-supplementary.pdf]

materials MDPI

Supplementary Materials

1. Material Preparation

Fluorine-doped Tin Oxide (FTO) with a sheet resistance of ca. 9 Ω sq^{-1} was purchased from, along with Formamidinium Iodide (FAI) and methylammonium chloride (MACl), Xi'an Polymer Light Technology Crop(xi'an,China). PbI_2 (99.8%), bis (trifluoromethane) sulfonimide lithium salt (Li-TFSI, 99%), 4-tert-butylpyridine (tBP, 96%), and CsI (99.99%) were supplied from Sigma-Aldrich, St. Louis, MO, USA . Tin (IV) oxide was purchased from Alfa Aesar((Thermo Fisher Scientific), Ward Hill, MA, USA)). DMF and DMSO were purchased from TCI (Tokyo Chemical Industry Co., Ltd.), Tokyo, Japan. Spiro-OMeTAD (purity: 99.5%) was purchased from Feiming Science and Technology Co., Ltd (Shenzhen, China. All chemicals were used as received without further treatment.

For now, this is the material that needs to be prepared, and the rest will be updated in the final result as machine learning predictions are made.

Table S1. The feature encoding method and description for 22 features in the dataset.

Num	Feature Name and Encoding Method	Feature Description
1	MA ratio (extracted from perovskite components)	Continuous numbers from 0 to 1
2	FA ratio (extracted from perovskite components)	Continuous numbers from 0 to 1
3	Cs ratio (extracted from perovskite components)	Continuous numbers from 0 to 1
4	Rb ratio (extracted from perovskite components)	Continuous numbers from 0 to 1
5	Pb ratio (extracted from perovskite components)	Continuous numbers from 0 to 1
6	Sn ratio (extracted from perovskite components)	Continuous numbers from 0 to 1
7	Br ratio (extracted from perovskite components)	Continuous numbers from 0 to 1
8	I ratio (extracted from perovskite components)	Continuous numbers from 0 to 1
9	Bandgap (predicted from perovskite components by Gok et al.'s model [1])	Continuous numbers
10	ETL (encoded by label-encoder)	Contains 141 alternatives
11	ETL-2 (encoded by label-encoder)	Contains 111 alterntives
12	Perovskite_deposition_procedure (encoded by label-encoder)	Contains 2 alternatives
13	Perovskite_deposition_method (encoded by label-encoder)	Contains 39 alternatives
14	Antisolvent (encoded by label-encoder)	Contains 34 alternatives
15	Precursor_solution (encoded by label-encoder)	Contains 88 alternatives

16	HTL (encoded by label-encoder)	Contains 244 alternatives
17	HTL_additive (encoded by label-encoder)	Contains 61 alternatives
18	ETL_Passivator (encoded by label-encoder)	Contains 33 alternatives
19	HTL_Passivator (encoded by label-encoder)	Contains 76 alternatives
20	Additives (encoded by label-encoder)	Contains 98 alternatives
21	Add_Cl (encoded by label-encoder)	Contains 2 alternatives
22	Type (encoded by label-encoder)	Contains 2 alternatives

Table S2. Stacking Ensemble Configuration.

Parameter	Value
Base Learners	SVR, Decision Tree, Random Forest, LightGBM, XGBoost, CatBoost
Meta-Learner	LightGBM
cv	5 (5-fold cross-validation)
Stack method	predict
passthrough	False
n_jobs	−1 (parallel computation)
Meta-Learner Params	learning_rate = 0.1, max_depth = 10, n_estimators = 500, num_leaves = 10

Table S3. Optimized Hyperparameters for Machine Learning Models.

Model	Key Parameters (Optimized)
SVR	Kernel = RBF, C = 10, ϵ = 0.5, Degree = 2
Decision Tree	Criterion = Friedman MSE, Max Depth = 10, Min Samples Leaf = 4
Random Forest	Estimators = 800, Max Depth = 20, Min Samples Split = 3
LightGBM	Learning Rate = 0.1, Estimators = 500, Max Depth = 10
XGBoost	Learning Rate = 0.02, Max Depth = 7, Estimators = 700
CatBoost	Depth = 5, Learning Rate = 0.1, Early Stopping = 2000
